# Efficacy of different pharmaceutical forms of *Curcuma longa* or curcumin in reducing oral mucositis severity and incidence in cancer patients: a systematic review and meta-analysis

**DOI:** 10.3389/fphar.2025.1560729

**Published:** 2025-04-02

**Authors:** Pedro de Padua G. Amatto, Lucas Chaves, Suzelei de Castro França, José Carlos Tavares Carvalho, Fabio Carmona, Ana Maria Soares Pereira

**Affiliations:** ^1^ Department of Biotechnology in Medicinal Plants, University of Ribeirão Preto, Ribeirão Preto, São Paulo, Brazil; ^2^ Ribeirão Preto Medical School, University of São Paulo, Ribeirão Preto, São Paulo, Brazil; ^3^ Department of Biological and Health Sciences, Federal University of Amapá, Macapá, Amapá, Brazil; ^4^ Botanical Garden of Medicinal Plants Ordem e Progresso, Jardinópolis, Brazil

**Keywords:** turmeric, anti-inflammatory agents, chemotherapy-induced mucositis, radiotherapy side effects, herbal medicine

## Abstract

**Background:**

*Curcuma longa* L. (turmeric, Zingiberaceae) has been traditionally used for its anti-inflammatory, wound-healing, and antimicrobial properties. These characteristics have made it a key component in managing inflammatory and ulcerative conditions like oral mucositis (OM). This study aimed to evaluate the effectiveness of various pharmaceutical formulations of *C. longa* or curcumin in reducing the OM severity, incidence and associated pain in patients undergoing chemotherapy and/or radiotherapy for cancer.

**Methods:**

This systematic review and meta-analysis of randomized clinical trials was conducted according to PRISMA guidelines, registered in PROSPERO (#CRD42024504111). Searches were performed in PubMed, Embase, and Cochrane databases. Studies comparing *C. longa* or curcumin with placebo in cancer patients experiencing oral mucositis, reporting outcomes such as the World Health Organization Oral Mucositis Grading Scale, pain scores (visual analogue scale), or OM incidence were included. Risk ratios and weighted mean differences with 95% confidence intervals were calculated using fixed- or random-effects models.

**Results:**

Six studies with 159 patients (mean age ∼50 years, 40% women) were included. *C. longa* extracts, curcumin, or nanocurcumin were administered in capsules, mouthwash, or gel formulations. The pooled analysis showed significant reductions in WHO scores and oral pain compared to placebo. OM incidence decreased by 6% overall, with a notable 37%-reduction observed in patients using curcumin-containing mouthwash during radiotherapy alone. Subgroup analyses revealed consistent benefits across all oncological treatments.

**Conclusion:**

*C. longa*, curcumin, or nanocurcumin in various formulations, effectively reduce OM severity and pain while curcumin-containing mouthwash reduced OM incidence in cancer patients undergoing treatment.

**Systematic Review Registration:**

identifier CRD 42024504111.

## Highlights

- *Curcuma longa* L. (turmeric) is used to treat skin and mucosal ailments.
- *C. longa* extracts decreased oral mucositis severity and pain in cancer patients.
- Mouthwash with curcumin decreased oral mucositis incidence of during radiotherapy.
- Benefits observed across chemotherapy, radiotherapy, and combined treatment modalities.
- Anti-inflammatory and wound-healing properties of *C. longa* contribute to its efficacy.

## 1 Introduction

Chemotherapy (CHT) and radiotherapy (RT) for cancer are frequently associated with significant adverse toxic effects. Among these, oral mucositis (OM) is one of the most prevalent and debilitating, affecting approximately 90% of patients undergoing such treatments (Sroussi et al., 2017; Villa and Sonis, 2016). OM is an acute inflammatory response characterized by ulceration of the mucosal and submucosal layers of the oral epithelium, leading to symptoms that range from localized pain to systemic complications such as anorexia, dehydration, and dysphagia (Lalla et al., 2019). The inflammatory and ulcerative nature of OM impacts the quality of life and increases the risk of secondary infections such as bacteremia (Facchini et al., 2012; Gamis et al., 2000; Kim et al., 2012; Lee and Galloway, 2022).

While multiple management strategies have been proposed, the burden of OM underscores the urgent need for effective interventions to prevent and treat it. Current treatments for OM, such as cryotherapy, benzydamine mouthwash, and palifermin, show varying degrees of efficacy but are associated with side effects or high costs. *Curcuma longa* (turmeric), a member of the Zingiberaceae family, provides a promising alternative due to its well-documented anti-inflammatory and wound-healing properties, as well as its favorable safety profile. Several studies suggest that its bioactive compounds can modulate inflammatory cytokines and accelerate mucosal regeneration, making it a viable complementary approach for OM management (Arun et al., 2020; Khafif et al., 2009; van’t Land et al., 2004; Zlotogorski et al., 2013).

In fact, systematic reviews and meta-analyses have shown that *C. longa* and/or curcumin decrease OM incidence and severity, (Dipalma et al., 2024; Wu et al., 2024; Zhang et al., 2021), but the routes, pharmaceutical forms, and dosages varied considerably. Understanding the effects of various pharmaceutical forms of *C. longa* and curcumin on the incidence and progression of OM is critical.

To address this gap, we conducted a systematic review and meta-analysis to compare the efficacy of different pharmaceutical forms of *C. longa* or curcumin in reducing the incidence and severity of OM in patients undergoing cancer treatment.

## 2 Methods

This article was based on Preferred Reporting Items for Systematic Reviews and Meta-Analyses (PRISMA) Guidelines (Page et al., 2021). A protocol was registered on the PROSPERO platform (CRD 42024504111).

### 2.1 Inclusion and exclusion criteria

This systematic review included only randomized clinical trials (RCTs) comparing *C. longa* with placebo in patients with OM during CHT or RT and which reported: at least one among these outcomes: (a) the World Health Organization (WHO) Oral Mucositis Grading Scale (Sonis et al., 1999), (b) a pain score (visual analogue scale), or (c) the incidence of OM; and well-defined *C. longa*- or curcumin-containing systemic or topical pharmaceutical forms (solutions, mouthwashes, tinctures, capsules, gels, creams and others). These formulations may differ in bioavailability, application convenience, and effectiveness in reducing OM severity and pain. Studies without a control group, which studied mixtures of medicinal plants, which did not report the outcomes of interest, and which studied patients without cancer, were excluded. Preclinical studies, case reports, observational studies, and articles not written in English were also excluded.

### 2.2 Source of information and search strategy

Only articles published before 19 December 2023, were searched in the following databases: PubMed, Embase, and Cochrane. The initial publication date was not limited. The query search used in this study was constructed using the PICOTS (population, intervention, comparison, outcome, time, and study type) method and is presented in Box 1.

BOX 1 **| Simplified search query used in PubMed, Embase and Scopus databases.**

[(“mucositis” OR “cancer” OR “stomatitis” OR “chemotherapy” OR “radiotherapy”) AND (“curcumin” OR “curcuminoids” OR “curcuma longa” OR “turmeric” OR “curcumin” OR “curcuminoids”) AND (“placebo”)].

### 2.3 Article selection

Two independent and initially blind reviewers (PA and LC) conducted the screening of articles by reading titles and abstracts. The PICOTS acronym guided this step: Patients receiving CHT or RT with OM (P), who used *C. longa* or curcumin (I), compared with placebo (C), for at least 3 weeks of treatment (T), with assessments of the WHO scale, pain, or incidence (O), in RCTs (S). The entire process used the Rayyan software (Ouzzani et al., 2016). Then, the blinding was revealed, and the reviewers resolved any disagreements.

### 2.4 Data collection

The articles selected to be read in full were randomly assigned to either of the two reviewers (PG and LC). The retrieved data were recorded in a Google Sheets spreadsheet (Alphabet Inc., Mountain View, CA, United States).

### 2.5 Quality assessment

This stage was conducted by FC, who assessed the risk of bias and quality of each study using the Cochrane Risk of Bias 2 tool (RoB 2, The Cochrane Collaboration, Copenhagen, Denmark).

### 2.6 Statistical analysis

Binary endpoints were summarized using the Mantel-Haenszel (MH) fixed-effects model, reporting risk ratios (RR) and 95% confidence intervals (CI). Treatment effects for continuous outcomes were summarized using mean differences (MD) and standard deviations (SD) retrieved from all trials. When not reported, they were estimated. MDs were calculated by subtracting the post-treatment mean from the baseline mean. SDs were estimated as follows: (a) from SE by the formula SD = SE × √n, where n is the number of patients; (b) from CI, calculating the range between the lower and upper limits, then dividing it by (SD = (95% CI_upper_ – 95% CI_lower_) ÷ 3.92); or (c) from p-values, obtaining corresponding t values from a t distribution table, then calculating SE by dividing MD by the t value (SE = MD ÷ t), then proceeding as above. The tool by Amy Drahota and Elaine Bellor, based on the calculations provided in the Cochrane Handbook, was used to aid the process (Higgins et al., 2022). GraphPad Prism version 8.0.0 statistical software (GraphPad, LaJolla, CA, United States) was used to calculate MD and SE in cases where only weekly patient data were provided. Standardized mean differences (SMD) were used to analyze outcomes reported in different units. Review Manager 5.4 (Nordic Cochrane Centre, The Cochrane Collaboration, Copenhagen, Denmark) was used for statistical analysis. Heterogeneity was assessed with the Cochran Q test and I^2^ statistics; p-values < 0.10 and I^2^ > 50% were considered significant for heterogeneity. Fixed-effects models were used for endpoints with I^2^ ≤ 50% (low heterogeneity), while random-effects models were used for outcomes with I^2^ > 50% (high heterogeneity). A sensitivity analysis was done for each outcome, seeking to assess the impact of individual studies on the overall result of each endpoint.

## 3 Results

### 3.1 Study selection and characteristics

**FIGURE 1:**
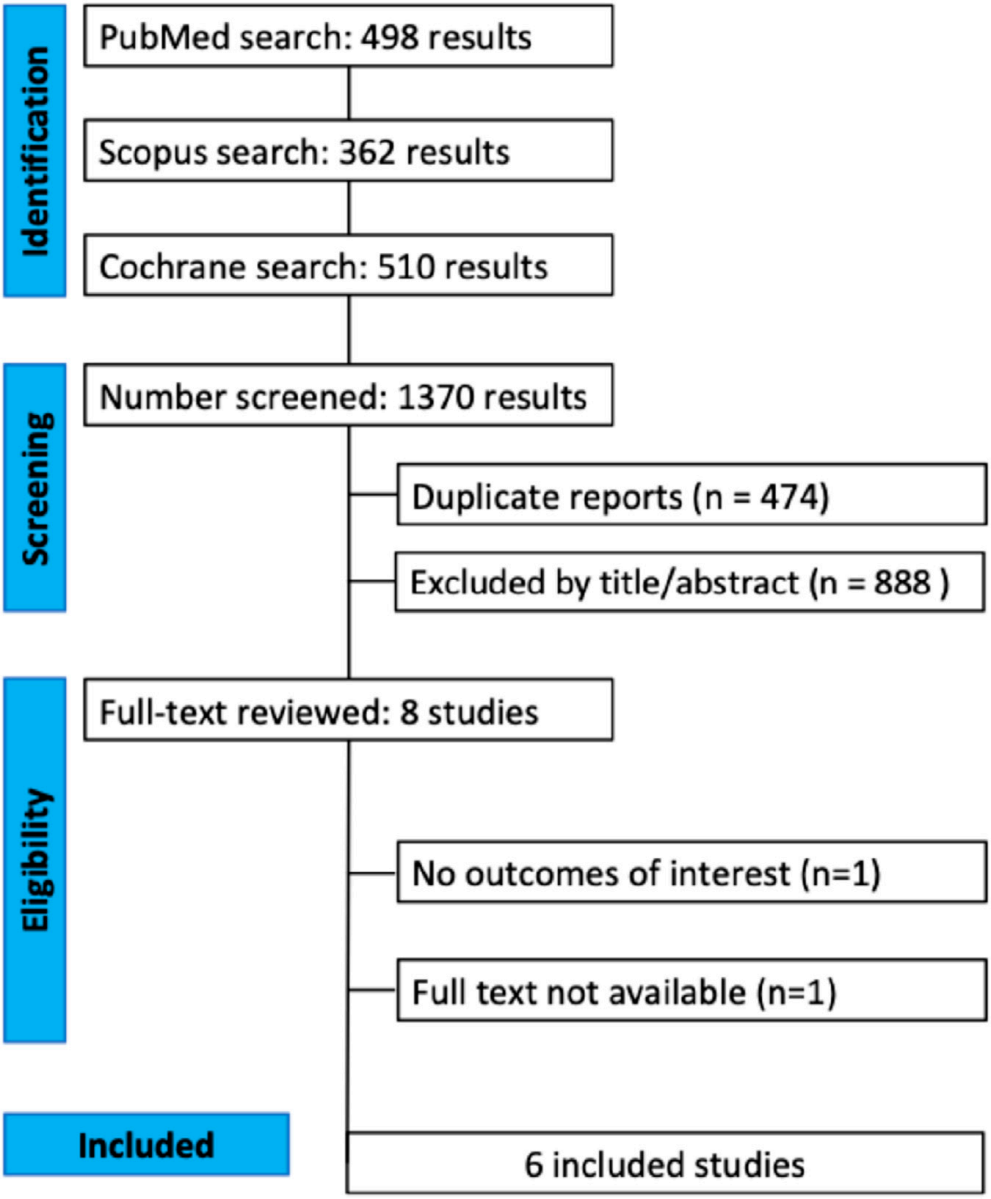
PRISMA flow diagram or study screening and selection.

The initial search yielded 1,370 articles. After removing duplicates and articles that were not of interest, eight (0.58% of the total) articles remained for full reading (Figure 1). One was excluded because the full text could not be obtained. Finally, six studies were included in the meta-analysis. All included studies had a 3- to 8-week follow-up time. *C. longa* or curcumin were used in four different pharmaceutical forms: (a) capsules containing SinaCurcumin^®^ (nano-micelles containing curcumin with the ability to disperse and dissolve in water) 40 or 80 mg/day (orally), (b) capsules containing BCM-95^®^ (purified extract containing 95% curcuminoids plus the essential oil, DER 25:1) 1.0 or 1.5 g/day (orally), (c) a mouthwash containing 0.1% (w/v) curcumin (topically), and (d) a gel containing 0.5% crude dried hydroethanolic extract of *C. longa* roots (topically). Most patients had head and neck cancer (mainly oral cancer), while some others had rectal cancer and other types. The included articles had 159 patients, of which 60 (40%) were women. Their mean age was around 50 years. The lowest radiation dose absorbed was 50 Gy, and the CHT scheme used was cisplatin alone or associated with 5-fluorouracil. Most studies had a two-arm study design, and all were placebo-controlled (Table 1).

**TABLE 1:** Baseline characteristics of included studies.

Study	Design	Intervention	Follow-up	Patients IN/CON	Females	Mean age (years)	Treatment	Inclusion criteria	Outcomes	Main findings
Mansourian et al. (2015)	Double-arm RCT	Gel containing 0.5% crude dried hydroethanolic extract of *C. longa* roots	8 weeks	19/18	15/16	47/55	RT (minimum radiation dose of 50 Gy)	Adults with HNC, minimally 50% of patient’s oral cavity in radiation field	WHO scale, pain, and incidence	Reduced OM severity and size of oral lesions
		Route: topical								
Delavarian et al. (2019)	Double-arm RCT	Curcumin (nanocurcumin) 80 mg/day	6 weeks	15/14	7/6	62/56	RT (minimum radiation dose of 50 Gy)	Adults with HNC, minimally 50% of patient’s oral cavity in radiation field	WHO scale, and incidence	Reduced OM incidence and severity
		Route: oral								
Arun et al. (2020)	Double-arm RCT	*C. longa* extract (BCM-95^®^, 95% curcuminoids, DER 25:1) 1.5 g/day	8 weeks	30/31	15/18	n/r (30–80)	Post-operative RT or CHT+RT, or concurrent CHT [RT: Cobalt 60 (66 Gy); CHT: cisplatin (50 mg/m^2^)]	Adults with advanced HNC (only squamous cell carcinoma)	WHO scale, and incidence	Reduced OM incidence and severity
		Route: oral								
Kia (2021)	Double-arm RCT	Curcumin (nanocurcumin) 80 mg/day	7 weeks	25/25	10/12	n/r (∼56)	RT+CHT or CHT [RT: 60–70 Gy; CHT: cisplatin 30–50 mg plus 5-fluorouracil 640–750 mg]	Adults with HNC before starting CHT+RT or with other cancer types before starting CHT alone.	WHO scale, pain, and incidence	Reduced OM incidence and severity
		Route: oral								
Soni (2021)	Triple-arm RCT	*C. longa* extract (BCM-95^®^, 95% curcuminoids, DER 25:1) 1.0 or 1.5 g/day	6 weeks	20/20/20	1/2/2	40/46/45	RT+CHT [RT: 60 Gy; CHT: cisplatin 40 mg/m^2^]	Adults after radical surgery for oral carcinoma from any primary site, with no prior history of RT to the head-neck Region	WHO scale, pain, and incidence	Reduced OM incidence and severity
		Route: oral								
Ramezani et al. (2023)	Triple-arm RCT	1) Mouthwash containing 0.1% w/v curcumin	3 weeks	13/12/12	5/5/4	52/56/52	RT [RT: minimum 60 Gy)	Adults with mild to moderate radiation-induced OM (grade 1–3)	WHO scale, pain, and incidence	Reduced OM incidence and severity with the two treatments
		Route: topical								
		2) Curcumin (nanocurcumin) 40 mg/day								
		Route: oral								

### 3.2 Pooled analysis of all studies

#### 3.2.1 WHO scale

**FIGURE 2:**
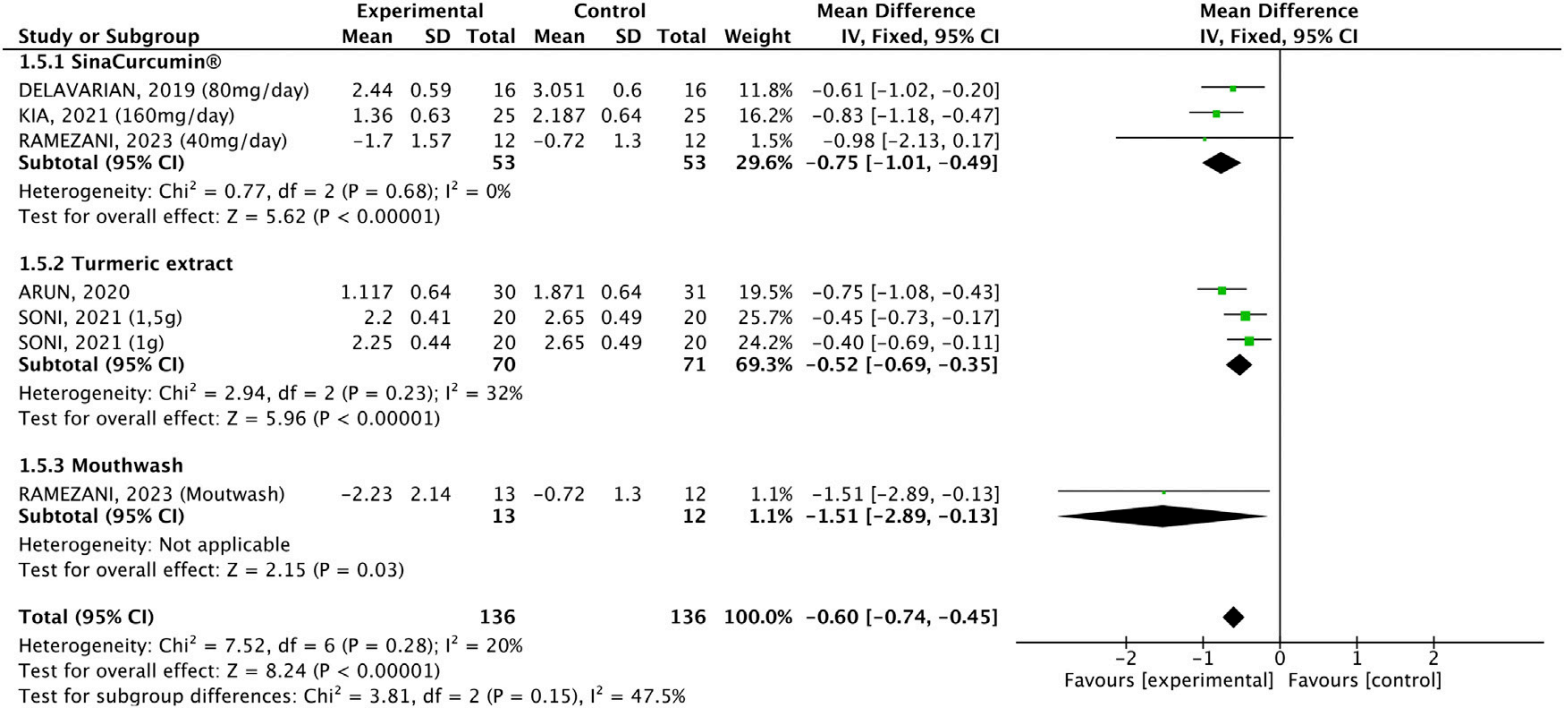
Forest plot of the efficacy of different pharmaceutical forms of *Curcuma longa* extract or curcumin on the WHO oral mucositis scale in cancer patients undergoing chemotherapy or radiotherapy, by pharmaceutical form.

**FIGURE 3:**
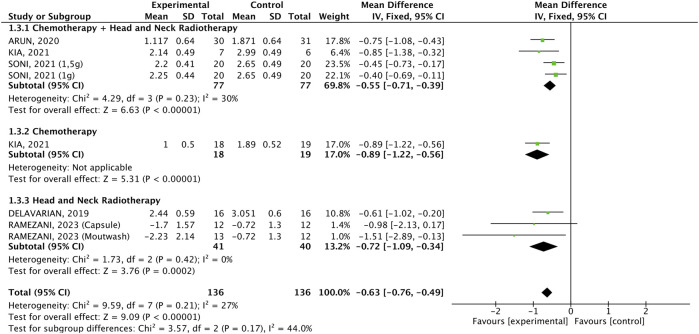
Forest plot of the efficacy of different pharmaceutical forms of *Curcuma longa* extract or curcumin on the WHO oral mucositis scale in cancer patients undergoing chemotherapy or radiotherapy, by cancer treatment.

Five studies compared 136 participants who received intervention with 136 who received placebo. The pooled result indicated that the WHO scale was significantly lower in patients receiving the interventions (WMD: −0.60%, 95% CI: −0.74, −0.45, p < 0.00001, Figure 2). All three formulations reduced the outcome (Delavarian et al., 2019, Soni et al., 2021, Kia et al., 2021, Ramezani et al., 2023, Arun et al., 2020). There was no significant heterogeneity (p = 0.28, I^2^ = 20%). Although the effect size for the mouthwash appears to be stronger, the subgroups did not differ significantly. Subgroup analysis according to the cancer treatment demonstrated that the intervention was equally effective in all three subgroups (WMD: −0.63, 95% CI: −0.76, −0.49, p < 0.00001, Figure 3), and there was no significant heterogeneity (p = 0.21, I^2^ = 27%).

#### 3.2.2 Pain

**FIGURE 4:**
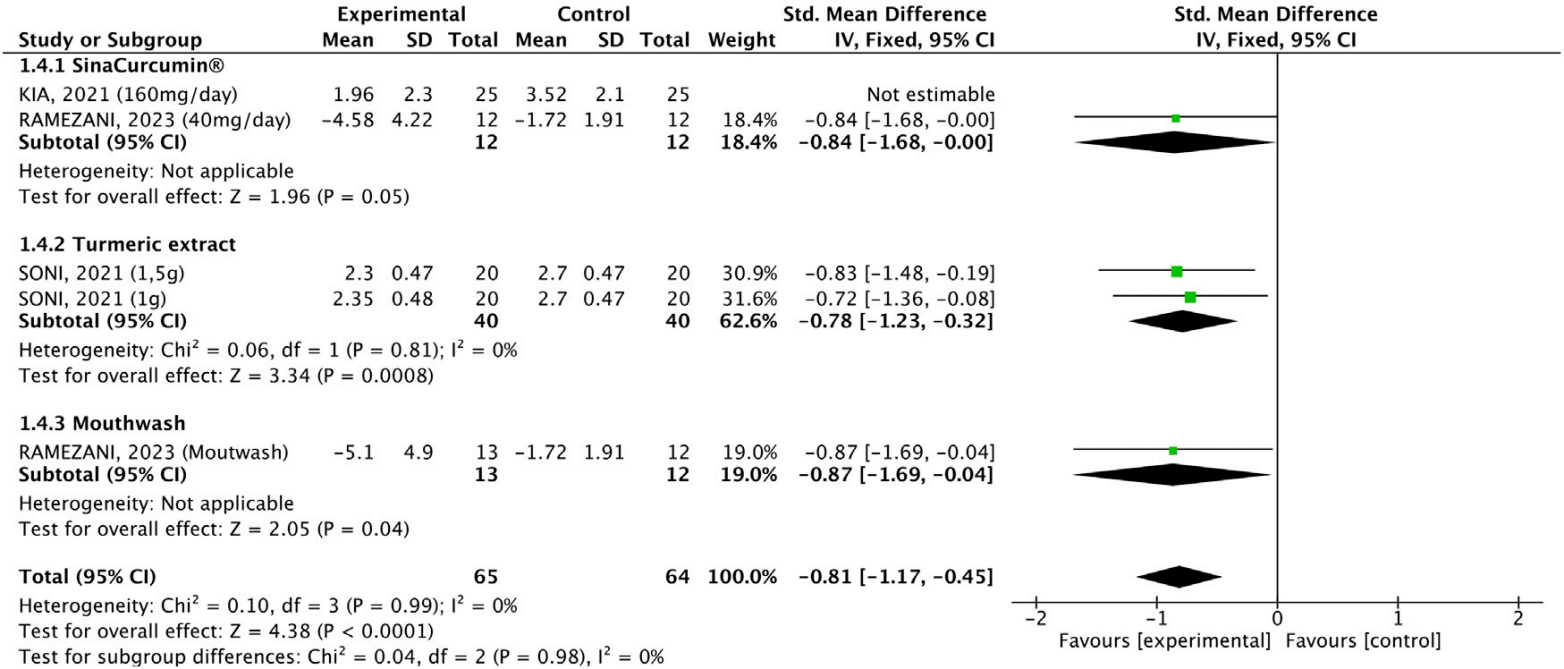
Forest plot of the efficacy of different pharmaceutical forms of *Curcuma longa* extract or curcumin on mucositis-related oral pain in cancer patients undergoing chemotherapy or radiotherapy, by pharmaceutical form.

**FIGURE 5:**
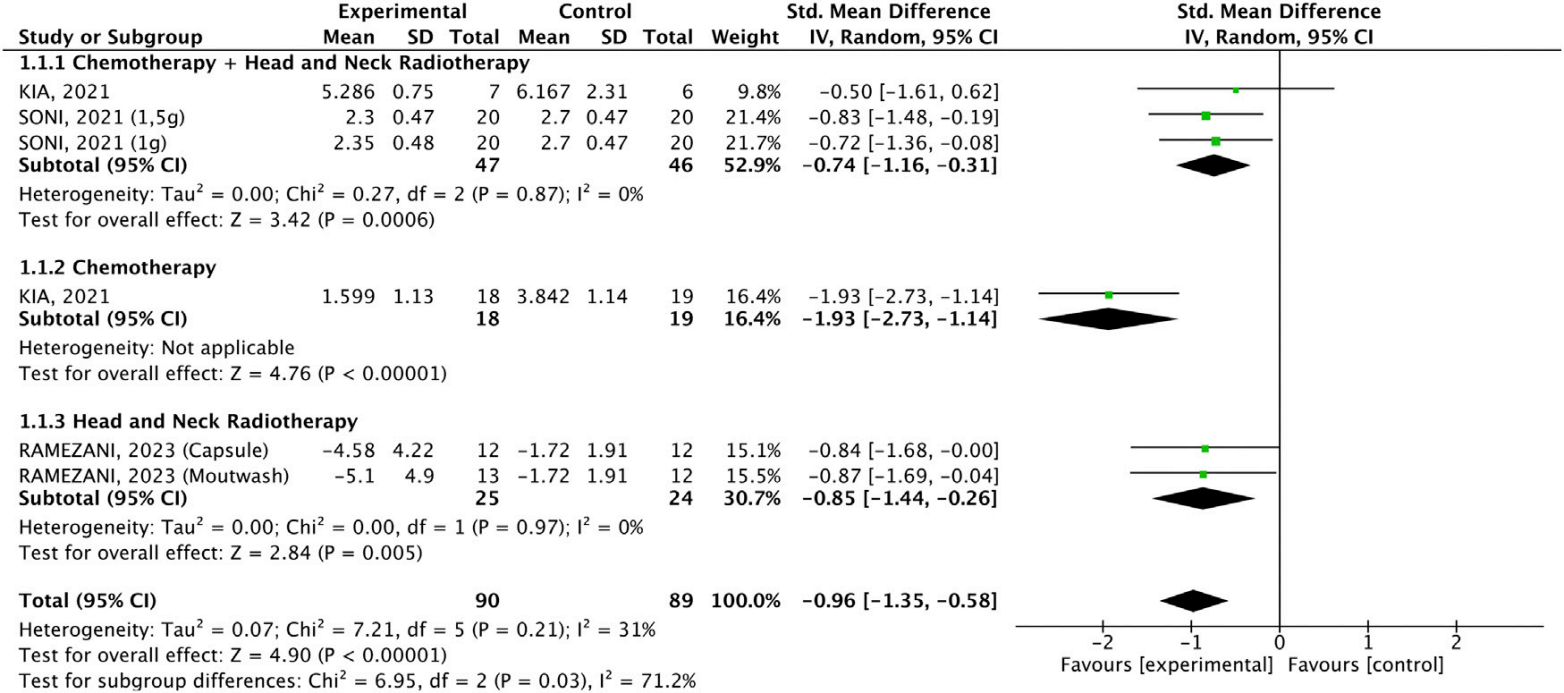
Forest plot of the efficacy of different pharmaceutical forms of *Curcuma longa* extract or curcumin on mucositis-related oral pain in cancer patients undergoing chemotherapy or radiotherapy, by cancer treatment.

Oral pain was assessed in five studies (90 participants who received *C. longa* or curcumin and 89 participants who used placebo) and was significantly lower in patients receiving the interventions (WMD: −0.81, 95% CI: −1.17, −0.45, p < 0.00001, Figure 4), and the three formulations equally decreased the outcome. There was no significant heterogeneity (p = 0.99, I^2^ = 0%). Subgroup analysis according to the cancer treatment demonstrated that the intervention was effective in all three subgroups (WMD: −0.96, 95% CI: −1.35, −0.58, p < 0.00001, Figure 5), but the effect was more prominent in the subgroup of patients undergoing CHT who received the SinaCurcumin^®^ (WMD: −1.93, 95% CI: −2.73, −1.14, p < 0.00001). There was no significant heterogeneity (p = 0.21, I^2^ = 31%).

#### 3.2.3 Incidence

**FIGURE 6:**
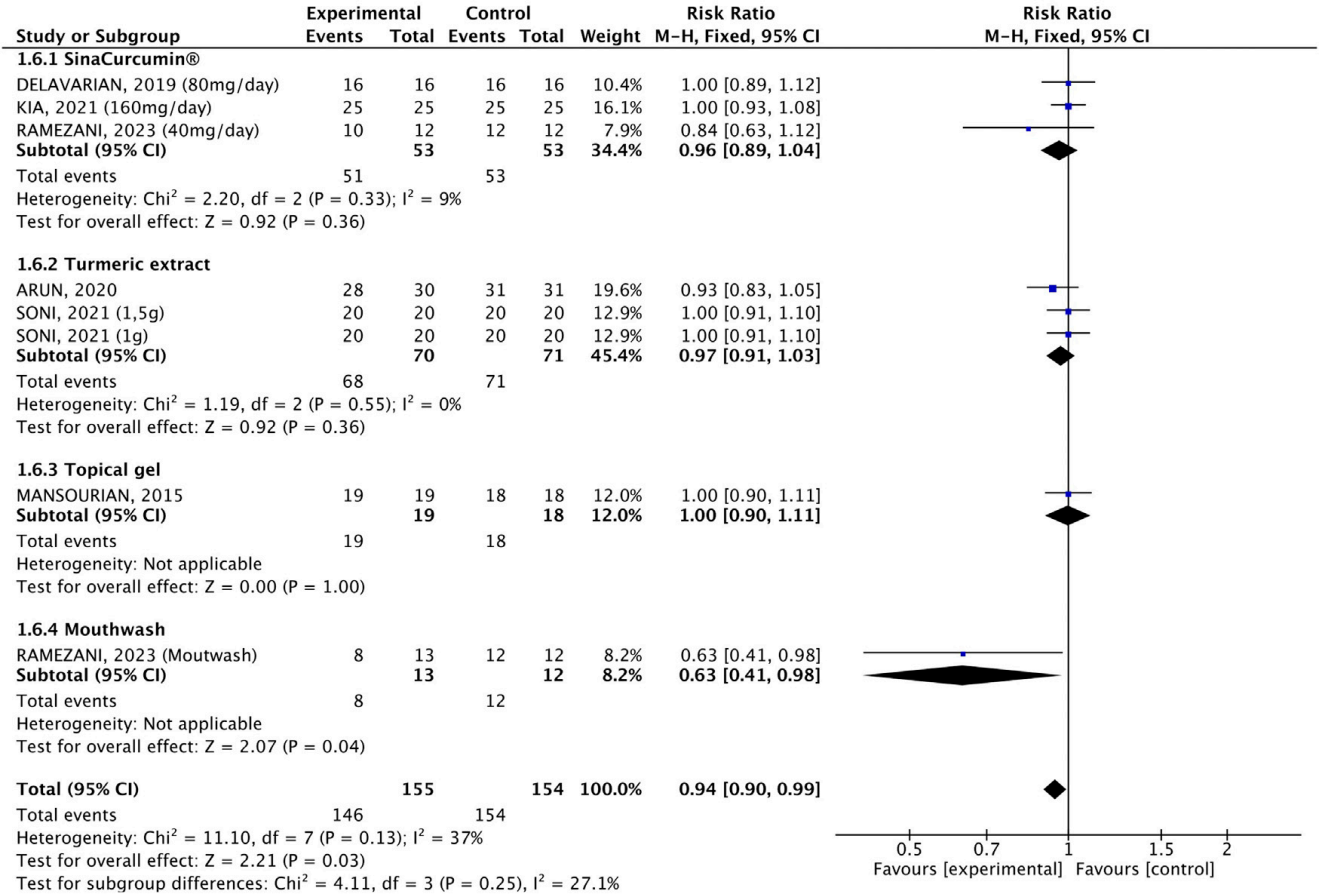
Forest plot of the efficacy of different pharmaceutical forms of *Curcuma longa* extract or curcumin on the incidence of oral mucositis in cancer patients undergoing chemotherapy or radiotherapy, by pharmaceutical form.

**FIGURE 7:**
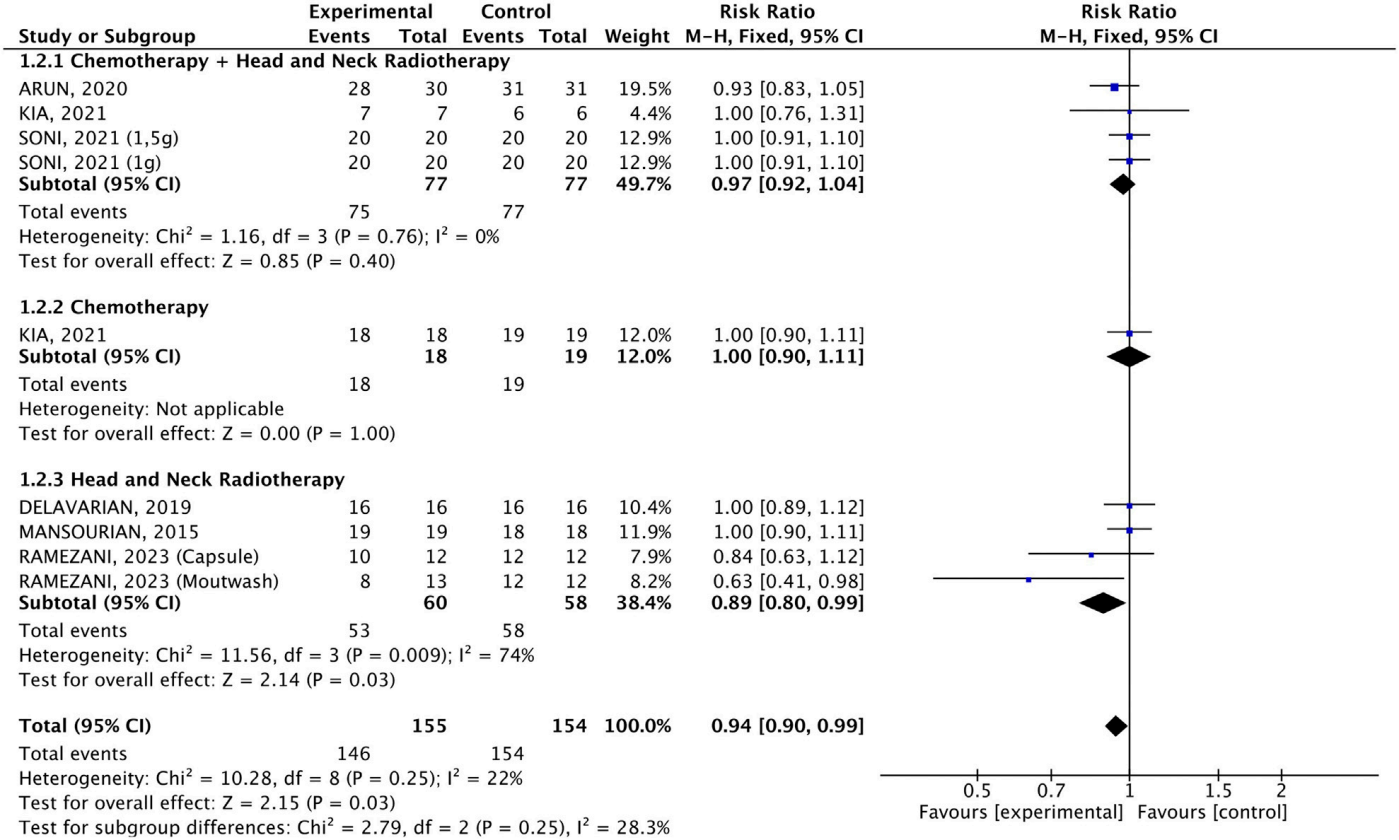
Forest plot of the efficacy of different pharmaceutical forms of *Curcuma longa* extract or curcumin on the incidence of oral mucositis in cancer patients undergoing chemotherapy or radiotherapy, by cancer treatment.

The incidence of OM was compared in 155 individuals who received *C. longa* or curcumin with 154 who received a placebo. The pooled analysis showed a 6%-reduction in OM incidence (RR 0.94, 95% CI: 0.90, 0.99, p = 0.03, Figure 6). The heterogeneity was not significant (p = 0.13, I^2^ = 37%), but the pooled result was driven mainly by one study, where the mouthwash containing curcumin led to a 37%-reduction in OM incidence (RR 0.63, 95% CI: 0.41, 0.98, p = 0.04) when compared to placebo, while the others were not statistically significant. In the subgroup analysis by cancer treatment, only patients undergoing RT and who received the mouthwash containing curcumin experienced an 37%-reduction in OM incidence compared to the placebo (RR 0.63, 95% CI: 0.41, 0.98, Figure 7), and this drove the pooled analysis, despite the low heterogeneity (p = 0.25, I^2^ = 22%).

### 3.3 Sensitivity analysis

For the WHO scale and pain outcomes, removing individual studies did not change the results. However, the positive result with the OM incidence outcome was driven mainly by the study by Ramezani et al., 2023, especially the group using mouthwash containing curcumin. Removing this study changes the pooled analysis to a negative result.

### 3.4 Quality assessment

**FIGURE 8:**
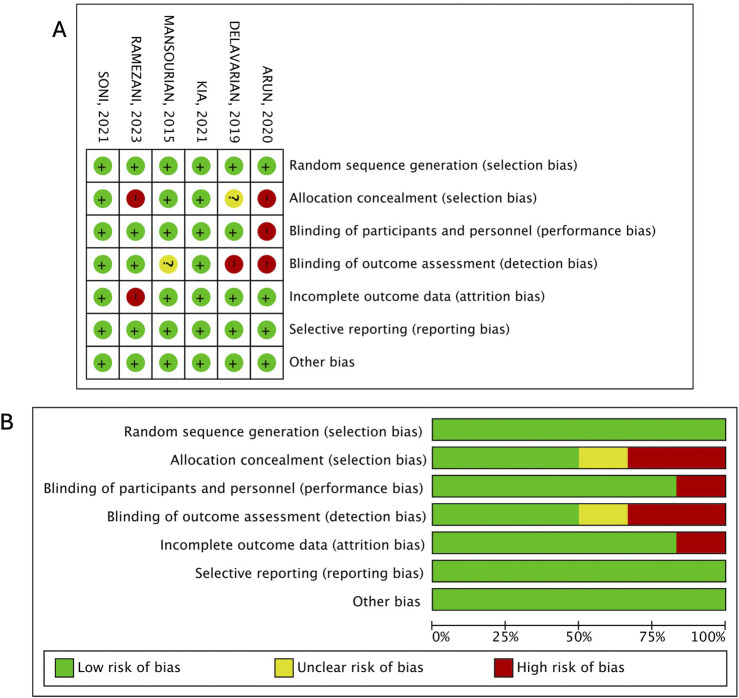
Risk of bias for each included study. **(A)**, risk of bias summary. **(B)**, risk of bias graph.

Of the six studies included in the meta-analysis, the study by (Arun et al., 2020) brought greater concern because it was at high risk of selection, performance, and detection biases. The study by (Ramezani et al., 2023) was at high risk of selection and attrition bias (>10% dropout rate), while the study by (Delavarian et al., 2019) was at high risk of detection bias. The other studies were at low risk of bias (Figure 8). By analyzing the funnel plots, we did not find evidence of publication bias (Supplementary Figures).

## 4 Discussion

This systematic review and meta-analysis of six studies involving over 300 patients with cancer demonstrated that *C. longa* extracts and curcumin, in different routes and pharmaceutical forms, significantly reduced OM severity, as measured by WHO scores, and oral pain, in a similar way. These benefits were seen in all oncological therapeutic modalities (CHT, RT, or CHT+RT), with slightly better outcomes in the CHT subgroup. Notably, mouthwash containing curcumin was the only formulation that decreased the incidence of OM in patients undergoing RT alone. In contrast, nanomicellar curcumin capsules demonstrated superior pain reduction in CHT-treated patients, likely due to improved systemic absorption. While all formulations were beneficial, the route of administration influenced the extent of the observed effects.

This is the first meta-analysis to systematically compare different pharmaceutical forms of *C. longa* and curcumin and assess their effectiveness across various cancer treatment modalities. Our findings align with previous meta-analyses (Zhang et al., 2021), which suggested a reduction in OM severity with *C. longa*, but extend this knowledge by addressing specific formulations and treatment subgroups.

Each formulation has distinct pharmacokinetic properties, advantages, and limitations. Oral curcumin capsules (nanomicellar or standard) deliver precise dosing and exert systemic anti-inflammatory and antioxidant effects, potentially benefiting mucosal healing beyond the oral cavity, but have a delayed onset of action due to the time required for absorption and systemic distribution, and limited direct contact with oral mucosa, potentially reducing its localized therapeutic effect. Curcumin-containing mouthwashes have a rapid onset of action because it is directly applied to the oral cavity, maximizing local prophylactic and therapeutic effects, but may require multiple daily applications for sustained effects, which may reduce patient adherence, may have poor palatability and cause patient discomfort due to taste or texture. Besides, the bioavailability of curcumin remains limited to the oral mucosa, with minimal systemic effects.

Topical curcumin gels provide prolonged mucosal contact, allowing sustained curcumin absorption at the site of inflammation, thus enhancing wound healing through direct anti-inflammatory and antimicrobial actions, besides easy application to localized lesions, but requires patient compliance and may be difficult in severe OM cases with widespread ulcerations. Curcumin release and absorption may vary, and systemic anti-inflammatory effects are lower than with oral administration.

The reduction in OM severity appears to be driven by *C. longa*’s anti-inflammatory, antioxidant, and analgesic properties. Its effects are likely mediated through inhibition of cyclooxygenase 2 and lipoxygenase enzymes, inhibition of the nuclear transcription factor kappa B (NF-κB), and suppression of inflammatory cytokines such as TNF-α, IL-1, IL-6, and IL-8 (Khafif et al., 2009; Zlotogorski et al., 2013). Additionally, *C. longa* promotes wound healing through re-epithelialization, macrophage migration, neovascularization, and antibacterial activity (Arun et al., 2020; van’t Land et al., 2004).

Interestingly, the benefit of the curcumin mouthwash in reducing OM incidence was investigated only in patients undergoing RT. This finding resonates with preclinical studies where curcumin reduced radiation-induced dermatitis in mice by modulating TGF-β pathways (Jagetia and Rajanikant, 2012) and a *C. longa* extract protected against 5-fluorouracil-induced oral mucositis in hamsters (Araújo et al., 2022). In a pilot study, this same mouthwash delayed the onset of OM in patients with head and neck cancer, similarly to a 0.15% benzydamine mouthwash (Shah et al., 2020).

Currently, there is a trend to use nanomicelles of natural substances to increase the availability in the body or site of action. The bioavailability of curcumin in the bloodstream is remarkably low after oral administration, which compromises its therapeutic efficacy. This limitation is attributed to low absorption, chemical instability, and rapid systemic elimination. Oral administration of curcumin fails to achieve adequate therapeutic results due to these challenges, resulting in minimal blood concentrations (Anand et al., 2007). An *in vivo* study demonstrated that low-dose nanocurcumin (20 mg/kg) exhibits comparable therapeutic effects to high-dose pure curcumin (400 mg/kg) (Szymusiak et al., 2016).

Other technologies can enhance curcumin’s bioavailability by up to 100-fold (Jamwal, 2018). Simpler strategies have also been employed, such as the association with piperine, which increases curcumin absorption up to 20-fold (Shoba et al., 1998), and the association of curcumin with *C. longa* essential oils, which increases curcumin absorption to 96% (Kiefer, 2007). The oral administration of a *C. longa* fresh root extract (rich in curcuminoids) to Wistar rats yielded higher plasma curcumin levels than an equivalent dose of purified (95.1%) curcuminoids extracted from dry *C. longa* roots, which failed to produce detectable curcumin in plasma. In humans, the absorption of total curcuminoids from the same extract was 46 times higher than equivalent doses of purified curcuminoids (Krishnakumar et al., 2015).

The cultivation of *C. longa* for extract production is well-established, and obtaining curcumin is an easy and cost-effective process. This makes the production of medicines from this medicinal plant feasible not only for developed countries but also for economically underdeveloped nations. The most used technologies (e.g., nano-micelles) are relatively simple and inexpensive, but even when these technologies are unavailable, a crude extract or even the powdered plant material can be used.

Although our results corroborate the findings by Zhang et al., 2021, these authors reported that *C. longa* only decreased the incidence of severe OM. Another recent meta-analysis reported the benefits of *C. longa* use in patients with head and neck cancer only in the fourth treatment week and not before or after (Wu et al., 2024). Although our study did not address the specific timing of effect onset, the mouthwash containing curcumin was effective as early as after 3 weeks.

These findings suggest that *C. longa* offers a promising therapeutic option for managing OM in cancer patients, with implications for prevention and treatment. Beyond its direct effects on OM, *C. longa* may also provide additional benefits such as enhanced wound healing and reduced systemic inflammation, potentially improving overall patient quality of life during cancer treatment (Kalluru et al., 2020). Given the ease of administration, especially with mouthwash formulations, *C. longa* could be incorporated into standard care protocols to mitigate OM in cancer patients.

Despite these promising results, our study has limitations. The evidence presented was restricted to the oral cavity, and the effectiveness of *C. longa* in preventing or managing mucositis beyond this region remains unclear. Additionally, the included studies varied in sample size, pharmaceutical forms, and dosage, which may limit the generalizability of our findings. Future research should address these gaps by standardizing formulations and exploring the effects of *C. longa* in larger, more diverse populations, including pediatric patients. Future studies should investigate the long-term effects of *C. longa* on chronic mucositis outcomes and its potential role in systemic inflammation management. Further exploration of specific formulations, such as mouthwashes, could provide insights into optimal dosing and administration frequency.

## 5 Conclusion

In conclusion, this meta-analysis demonstrates that *C. longa* extracts and curcumin, in different pharmaceutical forms, particularly curcumin-containing mouthwashes, significantly reduces OM severity and oral pain in cancer patients undergoing oncological treatment. These findings support its incorporation into clinical practice as a complementary therapy. Future research should focus on standardizing curcumin formulations, optimizing dosages, and expanding investigations to a broader oncological population to further validate these findings.

## Data Availability

The original contributions presented in the study are included in the article/Supplementary Material, further inquiries can be directed to the corresponding author.
