# Supplementary material for: Efficacy of different pharmaceutical forms of Curcuma longa or curcumin in reducing oral mucositis severity and incidence in cancer patients: a systematic review and meta-analysis
Source: Front Pharmacol. 2025 Apr 2;16:1560729. doi: 10.3389/fphar.2025.1560729 (PMC12000115; doi:10.3389/fphar.2025.1560729)
Supplement: Supplementary file 1 [file DataSheet1.docx]

# Supplementary figures

**Supplementary Figure 1.** Funnel plot of the efficacy of different pharmaceutical forms of *Curcuma longa* extract or curcumin on the WHO oral mucositis scale in cancer patients undergoing chemotherapy or radiotherapy, by pharmaceutical form.

**Supplementary Figure 2.** Forest plot of the efficacy of different pharmaceutical forms of *Curcuma longa* extract or curcumin on the WHO oral mucositis scale in cancer patients undergoing chemotherapy or radiotherapy, by cancer treatment.

**Supplementary Figure 3.** Forest plot of the efficacy of different pharmaceutical forms of Curcuma longa extract or curcumin on mucositis-related oral pain in cancer patients undergoing chemotherapy or radiotherapy, by pharmaceutical form.

**Supplementary Figure 4.** Forest plot of the efficacy of different pharmaceutical forms of Curcuma longa extract or curcumin on mucositis-related oral pain in cancer patients undergoing chemotherapy or radiotherapy, by cancer treatment.

**Supplementary Figure 5.** Forest plot of the efficacy of different pharmaceutical forms of Curcuma longa extract or curcumin on the incidence of oral mucositis in cancer patients undergoing chemotherapy or radiotherapy, by pharmaceutical form.

**Supplementary Figure 6.** Forest plot of the efficacy of different pharmaceutical forms of Curcuma longa extract or curcumin on the incidence of oral mucositis in cancer patients undergoing chemotherapy or radiotherapy, by cancer treatment.
